# Metagenome analysis reveals multi-kingdom gut microbiota as diagnostic markers for colorectal cancer

**DOI:** 10.3389/fmicb.2026.1805055

**Published:** 2026-07-01

**Authors:** Xuan Wang, Junyao Wang, Wei Chen, Jiaxin Sun, Jianye Li, Huimin Hu

**Affiliations:** 1 Department of Dermatology, Lianyungang Municipal Oriental Hospital, Lianyungang, China; 2 Department of Gastroenterology, Xiangyang Central Hospital, Affiliated Hospital of Hubei University of Arts and Science, Xiangyang, China; 3 Department of General Surgery, Lianyungang Municipal Oriental Hospital, Lianyungang, China; 4 State Key Laboratory of Pharmaceutical Biotechnology, Medical School, Nanjing University, Nanjing, China; 5 Jiangsu Key Laboratory of Molecular Medicine, Medical School, Nanjing University, Nanjing, China; 6 Department of Dermatology, The Affiliated Huai’an Hospital of Xuzhou Medical University and The Second People’s Hospital of Huai’an, Huaian, China

**Keywords:** colorectal cancer, diagnostic model, gut microbiota, MaAsLin2, metagenome

## Abstract

**Background:**

Colorectal cancer (CRC) is a major contributor to cancer-related morbidity and mortality globally. Emerging evidence suggests that gut microbiota plays a pivotal role in CRC development. However, the precise link between CRC and gut microbial dysbiosis remains poorly understood.

**Methods:**

In this study, we analyzed metagenomic datasets from 578 samples, sourced from five geographically distinct cohorts, including CRC patients and healthy controls from China, Austria, and Spain. This diverse cohort enabled us to investigate changes in the gut microbiome—bacteria, viruses, fungi, and archaea—in CRC patients across varying genetic and environmental contexts.

**Results:**

Our analysis led to the identification of 12 bacterial, 18 viral, and 1 fungal marker using a diagnostic model based on the gut microbiome. Notably, the multi-kingdom model, incorporating these markers, outperformed single-domain models in diagnostic accuracy. Integrating 24 microbial markers—comprising 9 bacterial, 14 viral, and 1 fungal marker—yielded an impressive AUROC of 0.911 for CRC diagnosis.

**Conclusion:**

This model demonstrated robust performance across four independent cohorts, confirming its potential as a highly accurate, non-invasive diagnostic tool for CRC.

## Introduction

Colorectal cancer (CRC) is the third most common cancer and the second leading cause of cancer-related deaths worldwide, with approximately 690,000 deaths annually (Joshi et al., 2017; Liu H. et al., 2022). CRC is a multifactorial disease, influenced by genetic and environmental factors, intestinal inflammation, and the gut microbiota (Lang et al., 2020). Its incidence continues to rise, driven in part by lifestyle and environmental changes that increasingly affect the gut microbiota (Wong and Yu, 2019; Fong et al., 2020).

Studies have shown that patients with CRC commonly exhibit gut microbial imbalances, with significant differences in microbial composition compared to healthy individuals. Notably, the bacterial communities in CRC patients are altered, with pathogenic species such as *Fusobacterium nucleatum*, *Peptostreptococcus anaerobius*, *Parvimonas micra*, and *Solobacterium moorei* becoming more prevalent. In contrast, beneficial bacteria like *Streptococcus thermophilus* and *Lactobacillus gallinarum* are reduced. These shifts suggest that changes in the gut microbiota contribute significantly to the onset and progression of CRC (Zhang et al., 2025).

In addition to bacterial imbalances, non-bacterial components of the microbiota, such as viruses, fungi, and archaea, are also altered in CRC patients. This complexity further complicates the exploration of the relationship between CRC and gut microbiota. The gut virome, which encompasses the full spectrum of viruses in the gut, differs significantly between CRC patients and healthy individuals. Notably, certain viruses, including bacteriophages and *Faecalibacterium viruses*, are more abundant in CRC patients (Zhang et al., 2022). These viruses may play a role in immune evasion by infecting intestinal cells or microbial communities. This interaction could help cancer cells within the tumor microenvironment evade immune surveillance, potentially accelerating tumor progression (Shen et al., 2021). The gut fungal communities of CRC patients are typically characterized by dysbiosis. While fungi such as *Saccharomyces cerevisiae* are typically low in abundance in healthy individuals, their levels, along with those of certain pathogenic fungi, are significantly elevated in CRC patients. This shift may be linked to alterations in intestinal inflammation and immune responses (Li et al., 2020). Fecal samples from CRC patients were significantly enriched in halophilic archaea and decreased in methanogenic archaea (Coker et al., 2020; Liu N. N. et al., 2022; Yang et al., 2021).

Early-stage colorectal cancer often presents no obvious symptoms and has an extremely long latency period, typically exceeding 10 years. When detected early, the 5-year survival rate for CRC is approximately 90% (Zhang et al., 2023; Dekker et al., 2019). The challenge of early CRC diagnosis underscores the urgent need for new technologies and biomarkers. Current screening methods, such as fecal occult blood tests (FOBT) and endoscopy, are limited by low sensitivity and specificity. Moreover, they are hindered by issues like poor patient compliance, discomfort, and high costs. Consequently, the development of more efficient diagnostic tools is critical. This need has fueled interest in new biomarkers, particularly those derived from gut microbiota, which could serve as complementary screening methods to improve diagnostic accuracy (Zgraggen et al., 2022; Franklyn et al., 2022).

In this context, biomarkers are essential for the early detection and diagnosis of CRC. Therefore, identifying relevant gut microbiota markers through cross-cohort studies has become a critical priority. Our study analyzed metagenomic datasets from five geographically diverse cohorts to assess the predictive power of both single- and multi-kingdom microbial communities. Using multivariate linear modeling with the MaAsLin2 algorithm and machine learning-based marker screening, we found that diagnostic models based on multi-kingdom microbial markers outperformed those using single-kingdom markers. Notably, a model incorporating just 24 multi-kingdom microbial features achieved a robust AUROC of 0.911, demonstrating stability across four independent cohorts. These findings highlight the unique microbiome signatures linked to CRC and emphasize the potential of multi-kingdom microbial markers as effective diagnostic tools.

## Methods

### Study design

The datasets were selected based on the following inclusion criteria: (1) fecal raw metagenomic sequencing data obtained from public databases; (2) case–control design with histologically confirmed colorectal cancer patients and healthy controls; (3) no antibiotic use, chemotherapy, or probiotic supplementation within the recent period; and (4) complete metadata. For model validation, we adopted a “from point to surface” strategy, where the test set was derived from a large cohort dataset in Shanghai, China, and the validation sets were progressively expanded from the same region to the same country but different regions, and finally to different countries, to systematically assess the model’s generalizability.

In order to obtain comprehensive information about the gut microbiota as well as to avoid biases arising from different data processing methods, we chose the sequence read archive (SRA) of raw sequencing metadata rather than the results of data processing from existing research platforms. Public datasets were retrieved from NCBI SRA database^1^ using keywords “colorectal cancer” and “human gut metagenome” (BioProject filter) through December 2024, yielding 84 projects. After excluding 16S rRNA amplicon sequencing studies and those lacking accessible raw data or matched metadata, 5 datasets met all inclusion criteria and were incorporated into our analysis. This study integrated 578 fecal shotgun metagenome sequencing samples from five different countries or regions. To demonstrate the universality of microbial signatures across regions, we divided the samples into training and validation cohorts. The training set comprised a large cohort from Shanghai, China (CHN_SH, PRJNA763023), including 100 controls and 100 CRC patients. The validation cohort were from Shanghai (China_SH, PRJNA731589), Inner Mongolia (China_NMG, PRJNA1138893), Spain (PRJNA961076) and Austria (PRJEB7774). The validation cohort comprised 197 controls and 181 CRC patients. All patients were diagnosed with CRC and had not received any treatment before stool sample collection.

### Sequencing data processing

The data were converted from SRA format to fastq format using the “fastq-dump” command in the SRA Toolkit. FASTQC (version 0.11.6) was employed to assess the quality-score distribution of sequencing reads (Specific parameters: time fastqc seq/*.gz -t 64). KneadData (version 0.12.0)^2^ was used to ensure the data contained high-quality microbial reads and was free of contamination. Low-quality reads, adapters, and human DNA contamination were removed using Trimmomatic to obtain clean reads (Specific parameters: -- trimmomatic-options “SLIDINGWINDOW:4:20 MINLEN:50” -- bowtie2-options “-- very-sensitive-dovetail”) (Bolger et al., 2014). Conda was used to establish the Kraken2 environment and to install Kraken2 (version 2.1.1) and Bracken (version 2.6.0) (Specific parameters: conda install kraken2 -c bioconda -y and conda install bracken = 2.6.0 -c bioconda). A custom Kraken2 database was constructed to encompass the following categories: bacteria, archaea, fungi, and virus using the Kraken2-build process. The preprocessed database was indexed by the Kraken2 tool (Specific parameters: time kraken2-build --build --threads 64 --db. /). Species abundance was estimated using the Bracken with a read length of 150 and k -mer length of 35 (Specific parameters: time bracken-build -d. / -t 64 -k 35 -l 150). The cleaned sequences were then annotated using Kraken2 based on bacterial, viral, fungal, and archaeal databases. Finally, Bracken was employed to estimate the relative abundance of bacteria, viruses, fungi, and archaea in each sample (Lu et al., 2022).

### Data analysis and model construction

Sample data were normalized using the phyloseq package, filtering out microbes with relative abundances below 0.01%. Stacked bar plots were generated in R using the ggtree and ggplot2 packages. MaAsLin2 was used to explore the correlation between CRC and the relative abundance of gut microbes. MaAsLin2 is a multivariate statistical model used to identify associations between microbial taxa and clinical metadata. The R code is: fit_data = Maaslin2 [input_data = df_input_data, input_metadata = df_input_metadata, output = “demo_output,” fixed_effects = c(“diagnosis”)]. Random forest analysis was performed using Hiplot software,^3^ a comprehensive web service for biomedical data analysis and visualization. The random forest was constructed with 1,000 decision trees and validated by 10-fold cross-validation. The Hiplot platform employs standard 10-fold stratified cross-validation rather than nested cross-validation. To assess multicollinearity among the 31 microbial features included in the model, we calculated variance inflation factor (VIF) and tolerance values using SPSS (version 21.0). A VIF threshold of 10 and a corresponding tolerance threshold of 0.1 were applied to identify highly collinear features. Features exceeding the VIF threshold or falling below the tolerance threshold were sequentially removed. ROC curves were plotted using MedCalc (version 15.2.2).

## Results

### Clinical characteristics of CRC and shotgun metagenome sequencing data processing

**Figure 1:**
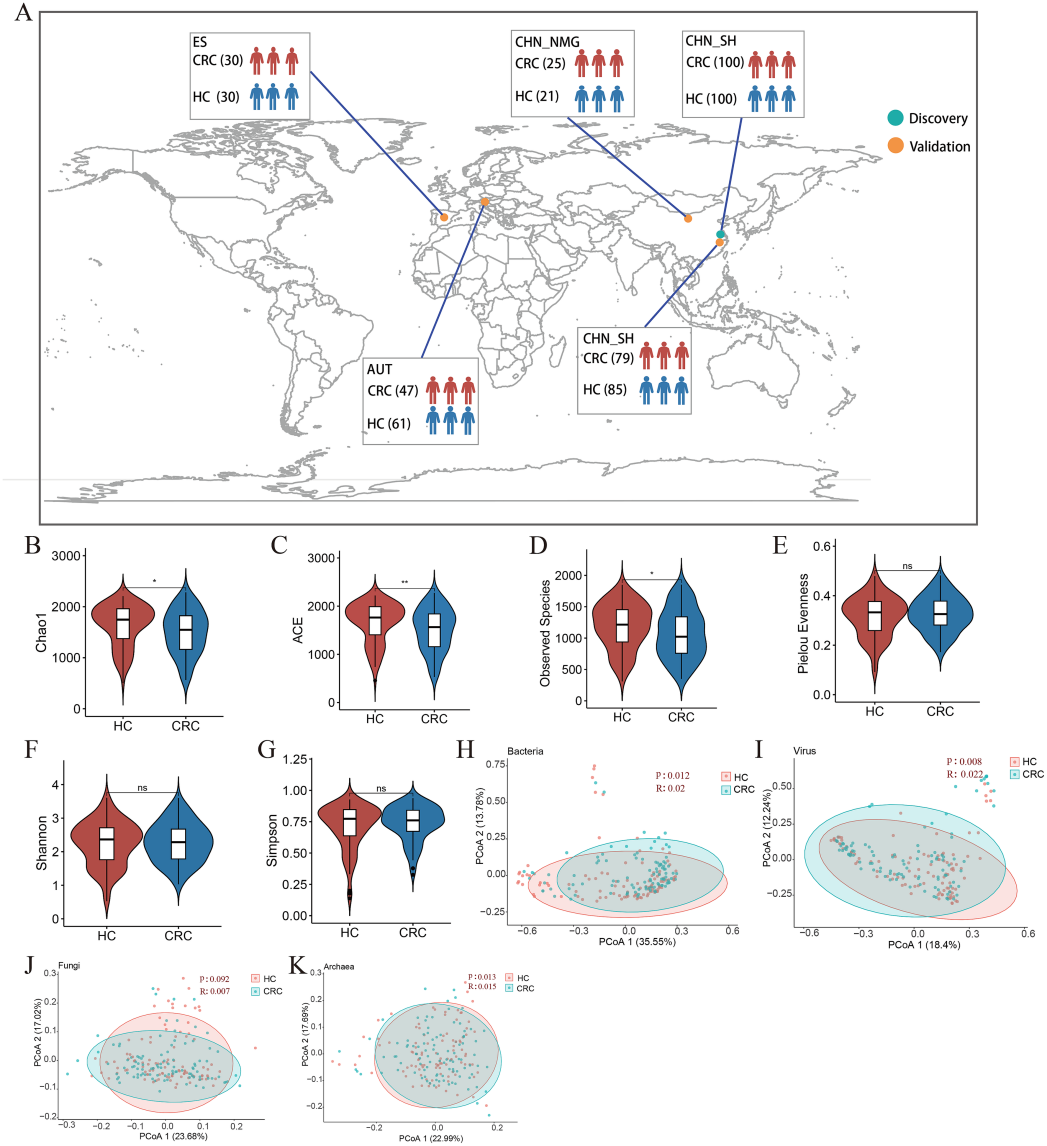
**(A)** The geographic distribution map of samples, which contains a total of 578 fecal shotgun metagenomic data from five datasets. HC represents healthy controls. CRC represents colorectal cancer. CHN_SH represents the cohort from Shanghai. CHN_NMG represents the cohort from Inner Mongolia, China. ES represents the cohort from Spain. AUT represents the cohort from Austria. **(B–G)** Differences in alpha diversity between CRC and controls. *p < 0.05; **p < 0.01; NS *p* > 0.05. **(H–K)** Principal coordinate analysis (PCoA) for bacteria, viruses, fungi, and archaea based on Bray-Curtis distances.

In this study, we collected 578 CRC samples, including 5 publicly available cohorts. The training dataset comprised 100 CRC patients and 100 controls (Shanghai, China). In the training cohort, no significant difference was found in age between CRC and HC groups (52.12vs 52.06) (*p* > 0.05). There was a significant gender difference between CRC and HC groups (*p <* 0.05). A significant gender imbalance was observed, with males being overrepresented in the CRC group relative to controls (64% vs. 48%, *χ*^2^ = 5.13, *p* = 0.023). Given the limited demographic information available in this database, this finding is presented as an exploratory observation rather than an established risk association. Non-quantitative faecal occult blood test (FOBT), Serum carcinoembryonic antigen (CEA) and cancer antigen 19–9 (CA 19–9) in CRC groups were significantly different from those in HC groups (*p <* 0.0001) (Table 1). To validate microbial markers that could be replicated for the diagnosis of patients with CRC, the independent validation datasets contained geographically more widely distributed samples with more complex genetic backgrounds, including 181 patients with CRC and 197 healthy controls from three countries (China, Austria, and Spain) (Figure 1A). To reduce technical bias in bioinformatics analysis, all raw shotgun sequencing data were processed using a uniform standard.

**Table 1:** Clinical characteristics in CRC and HCs.

Characteristics	Control	CRC	*P*-value
All participants (*n*)	100	100	N/A
Age	52.06 ± 13.89	52.12 ± 13.71	0.969^(a)^
Sex			0.023
Female	52	36	
Male	48	64	
Non-quantitative FOBT			< 0.0001
Negative	99	58	
Positive	1	42	
Serum CEA (mg/L)			< 0.0001
≤5.9	99	69	
>5.9	1	31	
Serum CA19-9 (kU/L)			< 0.0001
≤37	99	82	
>37	1	18	

CRC, colorectal cancer; FOBT, fecal occult blood test; N/A, not available. *P*-values calculated by two-tailed unpaired Student's *t-*test (a) or Pearson's Chi-square test. *P* < 0.05 considered statistically significant.

### Characteristics of gut microbiome in CRC

We first evaluated changes in alpha diversity in CRC patients compared with healthy controls. Microbial richness was significantly reduced in CRC patients according to Chao1, ACE, and Observed Species indices (Chao1, *p <* 0.05; ACE, *p <* 0.01; Observed Species, *p <* 0.05) (Figures 1B–D). However, according to Pielou Evenness, Shannon, and Simpson indices, there was no significant difference in microbial evenness between CRC patients and healthy controls (*p* > 0.05) (Figures 1E–G). We calculated beta-diversity indices across groups based on Bray-Curtis distances and plotted principal coordinate analyses (PCoA). The results showed that there were significant differences in bacteria, viruses and archaea between the healthy control and CRC groups. However, we did not observe significant differences between the two groups in fungi (Figures 1H–K). These results collectively suggest that there are distinct gut microbial communities characteristics between CRC and controls.

**Figure 2:**
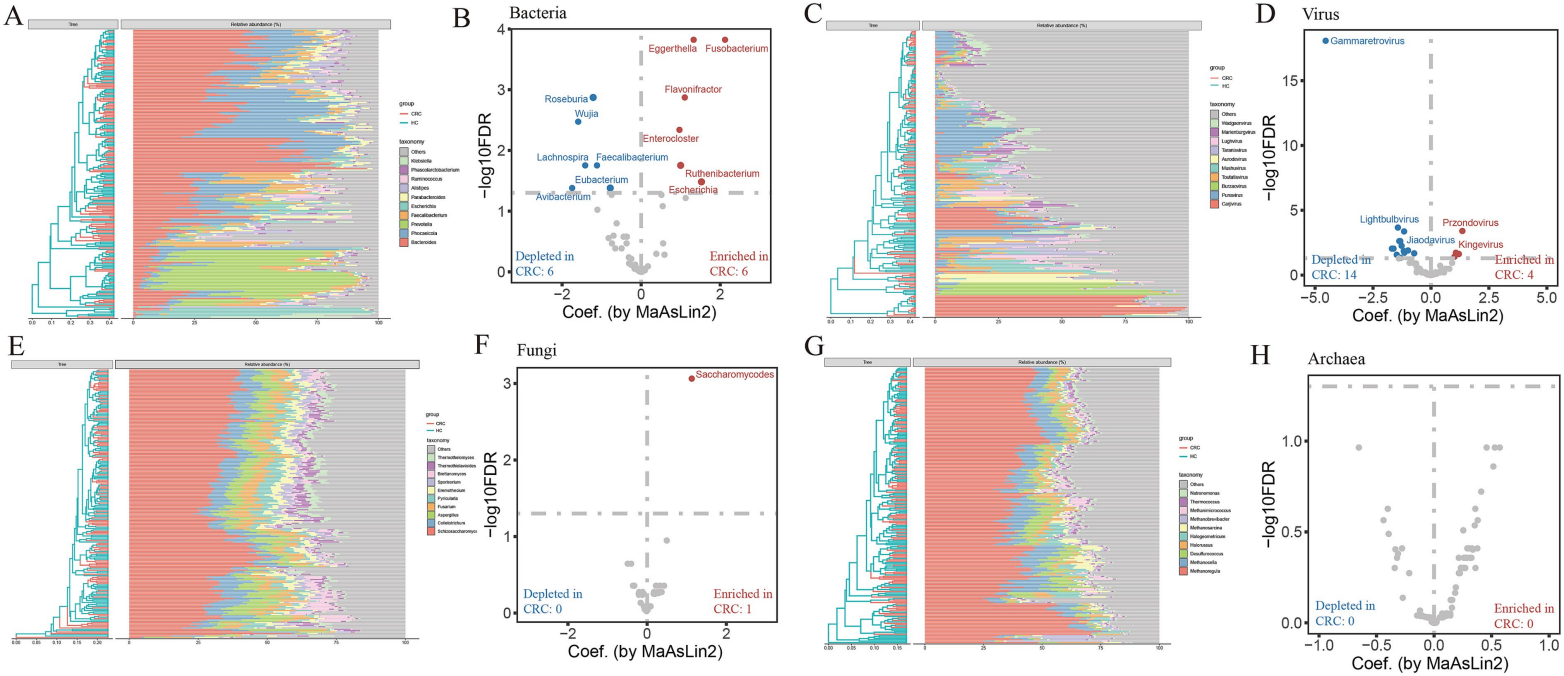
**(A)** Stacked bar plot of gut bacteria composition at the genus level. **(B)** Volcano plots show the associations between gut bacteria and CRC calculated by MaAsLin2. **(C)** Stacked bar plot of gut viruses composition at the genus level. **(D)** Volcano plots show the associations between gut viruses and CRC calculated by MaAsLin2. **(E)** Stacked bar plot of gut fungi composition at the genus level. **(F)** Volcano plots show the associations between gut fungi and CRC calculated by MaAsLin2. **(G)** Stacked bar plot of gut archaea composition at the genus level. **(H)** Volcano plots show the associations between gut archaea and CRC calculated by MaAsLin2.

MaAsLin2 was used to identify the correlation between CRC and gut microbiota, and multivariate regression models were run to explore the correlation between the abundance of gut microbiota and CRC diagnosis. A total of 12 gut bacterial markers, 18 gut viral markers and 1 gut fungal marker were identified. No gut archaeal biomarkers associated with CRC diagnosis were detected. Compared to healthy controls, 11 microbial genus were significantly enriched in the CRC groups, including *Fusobacterium nucleatum*, *Eggerthella lenta*, *Flavonifractor plautii*, *Enterocloster lactis*, *Ruthenibacterium lactatiformans*, *Escherichia coli*, *Przondovirus*, *Oryzopoxvirus*, *Kingevirus*, *Delmidovirus*, and *Saccharomycodes cerevisiae*. In this study, the abundance of Saccharomycodes at the fungal level was significantly elevated in CRC patients, although the underlying mechanism remains unclear. Conversely, 20 microbial genus were significantly depleted, including *Roseburia intestinalis*, *Wujia massiliensis*, *Faecalibacterium prausnitzii*, *Lachnospira pectinoschiza*, *Avibacterium gallinaceum*, *Eubacterium rectale*, *Gammaretrovirus*, *Lightbulbvirus*, *Jiaodavirus*, *Wellingtonvirus*, *Alphacarmovirus*, *Taipeivirus*, *Toutatisvirus*, *Oengusvirus*, *Matervirus*, *Sedonavirus*, *Eponavirus*, *Jedunavirus*, *Brigitvirus*, and *Lughvirus* (Figures 2A–H). In the validation cohort, *Fusobacterium* and *Escherichia* were significantly enriched in CRC across 3 out of 4 datasets, while *Flavonifractor* and *Enterocloster* showed significant enrichment in CRC in 2 out of 4 datasets. *Roseburia* and *Faecalibacterium* were reduced in CRC across 3 out of 4 datasets. These indicate that the gut microbiota of CRC shows commonalities across different studies (Supplementary Figure 1).

### Multi-kingdom microbial features can enhance prediction accuracy

**Figure 3:**
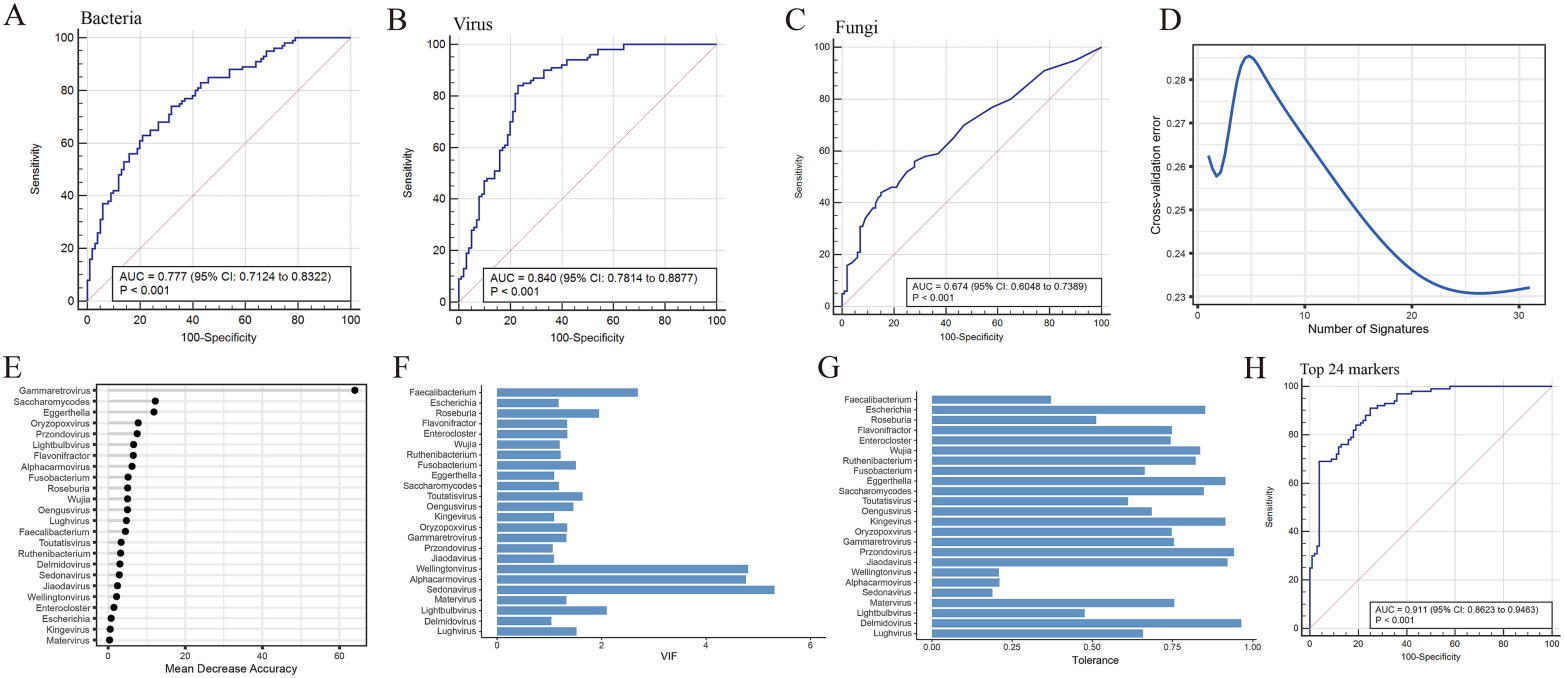
**(A)** ROC curve of gut bacteria in training cohort. **(B)** ROC curve of gut viruses in training cohort. **(C)** ROC curve of gut fungi in training cohort. **(D)** Results of 10-fold cross-validation. **(E)** Random Forest feature importance ranking for the 24 microbial biomarkers. **(F,G)** VIF and tolerance of the 24 microbial biomarkers. **(H)** ROC curve of multi-kingdom gut microbiota (bacteria, viruses and fungi) in training cohort.

We evaluated the predictive performance of gut microbial communities for CRC diagnosis by receiver operating characteristic (ROC) curves. In the training cohort, we first tested the accuracy of models using single-kingdom microbial biomarkers to distinguish CRC from healthy individuals. The results demonstrated that models constructed using the MaAsLin2 algorithm exhibited robust predictive performance: the area under the curve (AUC) value for the bacterial-based model reached 0.777 (95% CI: 0.7124–0.8322), the viral-based model achieved an AUC of 0.840 (95% CI: 0.7814–0.8877), while the fungal-based model achieved an AUC of 0.674 (95% CI: 0.6048–0.7389) (Figures 3A–C). This indicates that these characteristic microbial communities can serve as reference indicators for CRC diagnosis.

Given the diagnostic potential of all single-kingdom microbial features for CRC, we further explored the predictive capability of integrated multi-kingdom feature models. A total of 24 microbial biomarkers, including bacteria, viruses and fungi, were selected by the random forest classifier (Figures 3D,E). We tested the variables for multicollinearity by calculating tolerance and variance inflation factor (VIF). The results showed that the VIF was <10 and the tolerance of the variables was >0.1, indicating that there was no multicollinearity problem among all the independent variables (Figures 3F,G). We found that the model integrating multi-kingdom microbial features performed better in CRC diagnosis, with an area under the curve (AUC) of 0.911 (95% CI: 0.8623–0.9463), as compared with the single-kingdom microbial model (Figure 3H). Consistent with our hypothesis, integrating multi-kingdom features significantly enhanced CRC prediction, indicating that the combination of multi-kingdom microbial features possesses additive predictive value.

### Validating the reliability of multi-kingdom microbial combinations in independent cohorts

**Figure 4:**
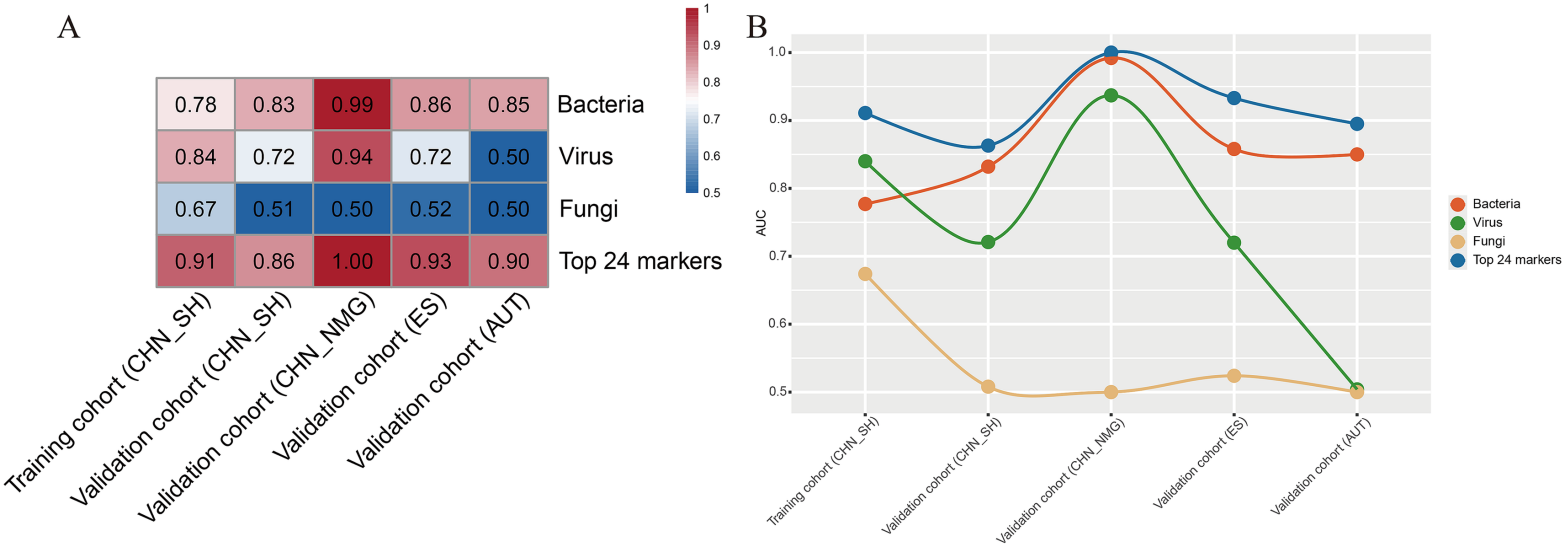
**(A,B)** The heatmap and curve graph shows the AUROC values in training and validation cohort. CHN_SH represents the cohort from Shanghai. CHN_NMG represents the cohort from Inner Mongolia, China. ES represents the cohort from Spain. AUT represents the cohort from Austria.

To further validate the diagnostic value of microbial communities, we conducted independent testing using four external validation cohorts from different regions and countries to confirm the model’s reliability. In the CHN_SH validation cohort, the AUC for the bacterial model was 0.832 (95% CI: 0.7660–0.8859), the AUC for the viral model was 0.721 (95% CI: 0.6458–0.7882), the AUC for the fungal model was 0.508 (95% CI: 0.4291–0.5870), and the AUC for the multi-kingdom microbial model was 0.863 (95% CI: 0.8011–0.9119). In the CHN_NMG validation cohort, the AUC for the bacterial model was 0.992 (95% CI: 0.9096–1.0000), the AUC for the viral model was 0.937 (95% CI: 0.8243–0.9874), the AUC for the fungal model was 0.500 (95% CI: 0.3490–0.6510), and the AUC for the multi-kingdom microbial model was 1.000 (95% CI: 0.9229–1.0000). In the validation cohort of ES, the AUC for the bacterial model was 0.858 (95% CI: 0.7435–0.9345), the viral model AUC was 0.720 (95% CI: 0.5891–0.8283), the fungal model AUC was 0.524 (95% CI: 0.3914–0.6550), and the AUC for multi-kingdom microbial model was 0.933 (95% CI: 0.8380–0.9815). In the validation cohort of AUT, the AUC for the bacterial model was 0.850 (95% CI: 0.7689–0.9117), the AUC for the viral model was 0.504 (95% CI: 0.4061–0.6017), the fungal model had an AUC of 0.500 (95% CI: 0.4024–0.5979), and the AUC for multi-kingdom microbial model was 0.895 (95% CI: 0.8217–0.9460) (Figures 4A,B). Therefore, we conclude that the performance of the multi-kingdom microbial prediction model significantly outperforms single-kingdom microbial prediction models.

## Discussion

Colorectal cancer (CRC) ranks as the third most prevalent and lethal malignancy globally, with a higher incidence in developed countries. In 2023, the United States is projected to see approximately 153,020 new CRC cases and 52,550 deaths attributed to the disease (Siegel et al., 2023). Most patients are diagnosed at an advanced stage, preventing curative surgical intervention. Early detection thus remains a major challenge in both clinical practice and research. Current diagnostic methods include fecal occult blood test, fecal immunochemical test (FIT), and multi-target stool DNA test, alongside visual inspection techniques such as colon capsule endoscopy and flexible sigmoidoscopy (Gude et al., 2023). However, stool-based tests are limited by sensitivity and specificity, while visual inspection methods face issues such as invasiveness, high cost, and poor patient compliance. There is, therefore, an urgent need for novel non-invasive diagnostic tools.

Previous studies have established a strong link between CRC and gut bacteria. *Fusobacterium nucleatum* and *Parvimonas micra* are significantly enriched in the intestines of CRC patients, with *Fusobacterium nucleatum* emerging as a key bacterial biomarker for early diagnosis, risk stratification, and outcome prediction (Wang et al., 2023). In contrast, research on viruses and fungi associated with CRC remains limited. In this study, we integrated metagenomic data from 578 stool samples across five geographically distinct cohorts to profile gut bacteria, viruses, fungi, and archaea in CRC patients. We then developed a diagnostic model based on multi-kingdom microbial biomarkers, offering a new theoretical framework and technical support for precise CRC diagnosis.

Through comprehensive analysis of multi-kingdom microbial community characteristics, we identified significant changes in gut microbial diversity among CRC patients. Specifically, the abundance of beneficial short-chain fatty acid (SCFA)-producing bacteria, such as *Roseburia*, *Wujia*, and *Faecalibacterium*, was markedly reduced in the CRC group. *Roseburia* is a Gram-positive, anaerobic bacterium with a slightly curved rod morphology and flagella-mediated motility. It exerts protective effects on the host by producing SCFAs and secondary bile acids, which regulate colonic peristalsis, immune responses, and inflammation (Tamanai-Shacoori et al., 2017). Kang et al. (2023) demonstrated that *Roseburia* enhances anti-PD-1 efficacy by promoting CD8 + T cell butyrate production. *Wujia* has been shown to correlate with hemoglobin levels and is enriched in the lower respiratory tract (LRT) microbiome (Liu et al., 2021). *Faecalibacterium*, a strictly anaerobic and oxygen-sensitive Gram-positive bacillus, is present in 85% of human gut samples and supports the differentiation of CD4 + T cells (Duncan et al., 2002; De Filippis et al., 2020). A study from Kenyan further indicated that *Faecalibacterium* is depleted in CRC patients, a depletion linked to disrupted glutamate metabolism (Obuya et al., 2022).

Conversely, bacterial taxa associated with inflammation and tumor progression, such as *Fusobacterium*, *Eggerthella*, *Flavonifractor*, *Enterocloster*, *Ruthenibacterium*, and *Escherichia*, was found to be elevated in CRC patients. *Fusobacterium,* a Gram-negative anaerobic bacterium typically found in the oral cavity, has been implicated in CRC pathogenesis. Emerging evidence suggests that *Fusobacterium* in the gut may originate from oral seeding, followed by colonization of the intestinal lumen (Komiya et al., 2019). It promotes carcinogenesis through multiple mechanisms, including enhancing cell proliferation, accelerating cell invasion, inducing chronic inflammation, and facilitating immune evasion (McIlvanna et al., 2021; Omran et al., 2025). *Eggerthella*, an anaerobic Gram-positive bacillus, is a normal component of the human gut microbiota (Wade et al., 1996). However, intestinal barrier dysfunction in CRC patients may lead to *Eggerthella* translocation from the gut to the bloodstream, resulting in bacteremia (Woerther et al., 2017). *Flavonifractor plautii*, a Gram-positive bacterium from the genus *Clostridium*, is significantly enriched in the CRC gut. It serves as a key discriminant taxon, positively correlating with intestinal inflammatory markers (Yang et al., 2021), and contributes to the inhibition of Th2 immune responses by activating Treg and Th1 cells (Ogita et al., 2020). *Enterocloster,* following antibiotic treatment, can trigger the downregulation of mucosal addressin cell adhesion molecule 1 (MAdCAM1) through re-colonization, thereby inducing the migration of immunosuppressive cells from the gut to tumors (Fidelle et al., 2023). *Ruthenibacterium* is consistently enriched in the CRC gut, making it a promising candidate for non-invasive biomarkers (Bakir-Gungor et al., 2024; Trivieri et al., 2020). Finally, *Escherichia,* particularly abundant in recurrent right-sided CRC, reshapes the tumor microenvironment into an immunosuppressive, lipid-overloaded state (de Oliveira Alves et al., 2024).

Research on the relationship between viruses and CRC remains limited. However, cohort studies have reported a significant increase in the diversity of gut phage communities in CRC patients (Nakatsu et al., 2018; Chen et al., 2023). Phages may contribute to inflammation and carcinogenesis by altering microbial balance or inducing bacterial lysis, which promotes the proliferation of pro-inflammatory and carcinogenic bacteria (Gao et al., 2021). Latent DNA viruses can also disrupt the cell cycle and modulate CRC-related regulatory proteins and signaling pathways, such as the MAPK/AP1 pathway (Marongiu and Allgayer, 2022; Ivancevic et al., 2024). While much of the research on *Gammaretrovirus* has focused on prostate cancer, *Jiaodavirus* and *Przondovirus* have been linked to *Klebsiella pneumoniae* infections, and *Alphacarmovirus* is associated with herbaceous plants (Sfanos et al., 2011; Peters et al., 2024; Fang et al., 2022; Gudeta et al., 2023). Interestingly, the abundances of *Lightbulbvirus*, *Wellingtonvirus*, *Alphacarmovirus*, *Taipeivirus*, and *Toutatisvirus* were lower in CRC patients than in healthy individuals. In contrast, *Oryzopoxvirus*, *Kingevirus*, and *Delmidovirus* were more abundant in CRC patients. However, the role of these viral taxa in CRC remains underexplored.

Cross-cohort validation confirmed the potential clinical applicability of the multi-kingdom microbial biomarkers identified in this study. Despite notable differences in dietary patterns and genetic backgrounds across cohorts, the multi-kingdom microbial model demonstrated stable diagnostic performance. In the Inner Mongolia (China) cohort, the area under the receiver operating characteristic curve (AUROC) reached 1.000, while in the Spanish and Austrian cohorts, it was 0.933 and 0.895, respectively. These results suggest that the identified biomarkers reflect common features of CRC-associated gut dysbiosis, rather than being influenced by regional dietary or genetic factors.

Despite significant progress, this study has several limitations. First, the cohorts were not representative of diverse ethnicities and age groups, and the generalizability of the biomarkers requires validation in a broader population. Second, while the study focused on microbial community composition and its diagnostic value using metagenomic data, it did not establish causal relationships between the biomarkers and CRC through *in vitro* experiments or animal models. Finally, we were unable to identify effective diagnostic biomarkers for archaea, likely due to limitations in sample size or detection techniques. Future research should optimize detection methods to explore the role of archaea in CRC more comprehensively.

In conclusion, this study systematically characterized the multi-kingdom gut microbiota profiles and identified 24 microbial biomarkers for CRC with diagnostic value across cohorts. We constructed a highly efficient and stable non-invasive diagnostic model. Our findings overcome the limitations of single-kingdom microbiome research and provide novel tools for early CRC diagnosis, while deepening our understanding of CRC-related gut dysbiosis. With continued research into the mechanisms of multi-kingdom microbial interactions, future strategies to modulate gut microbiota composition hold great potential for enhancing CRC prevention and treatment, paving the way for precision medicine in CRC.

## Data Availability

The datasets presented in this study can be found in online repositories. The names of the repository/repositories and accession number(s) can be found in the article/Supplementary material.

## Footnotes

1
http://www.ncbi.nlm.nih.gov/sra/

2
http://huttenhower.sph.harvard.edu/kneaddata

3
https://hiplot.com.cn

## Supplementary material

SUPPLEMENTARY FIGURE 1 The commonalities of potential microbial markers in CRC across different studies. The validation cohorts were, respectively, from Shanghai, China **(A)**, Inner Mongolia, China **(B)**, Spain **(C)**, and Austria **(D)**.
