# Supplementary figures and images for: Metagenome analysis reveals multi-kingdom gut microbiota as diagnostic markers for colorectal cancer
Source: Front Microbiol. 2026 Jul 1;17:1805055. doi: 10.3389/fmicb.2026.1805055 (PMC13383993; doi:10.3389/fmicb.2026.1805055)

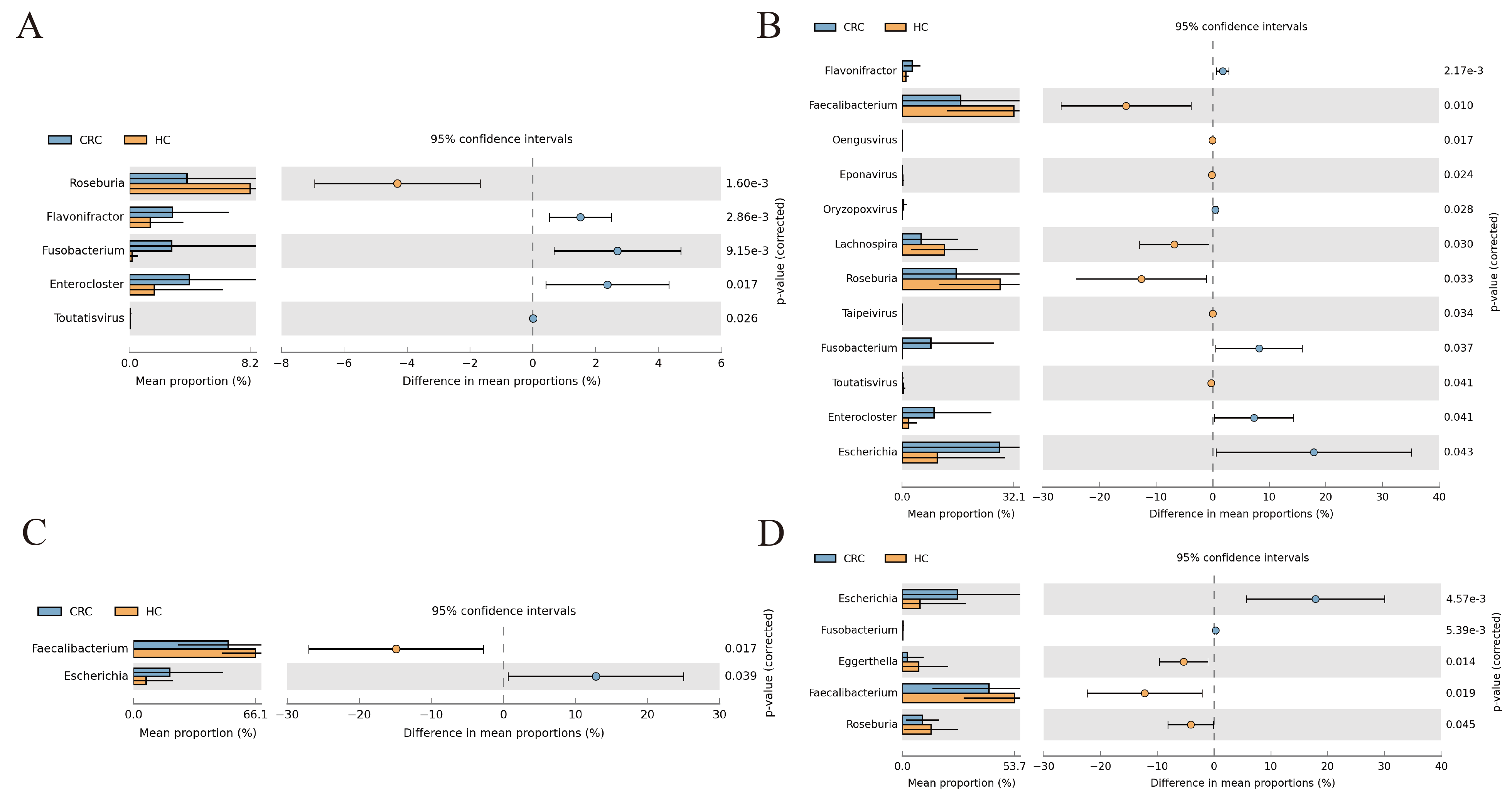

Supplement: SUPPLEMENTARY FIGURE 1 — The commonalities of potential microbial markers in CRC across different studies. The validation cohorts were, respectively, from Shanghai, China (A), Inner Mongolia, China (B), Spain (C), and Austria (D). [file Image_1.tif]
